# Anticipating sea‐level rise and human migration: A review of empirical evidence and avenues for future research

**DOI:** 10.1002/wcc.747

**Published:** 2021-11-12

**Authors:** Sem J. Duijndam, Wouter J. W. Botzen, Liselotte C. Hagedoorn, Jeroen C. J. H. Aerts

**Affiliations:** ^1^ Institute for Environmental Studies (IVM) Vrije Universiteit Amsterdam Amsterdam The Netherlands; ^2^ Utrecht University School of Economics (U.S.E.), Utrecht University Utrecht The Netherlands; ^3^ Risk Management and Decision Processes Center, The Wharton School University of Pennsylvania Philadelphia Pennsylvania USA; ^4^ Deltares Delft The Netherlands

**Keywords:** adaptation, migration, sea‐level rise, systematic literature review

## Abstract

Sea‐level rise (SLR) threatens millions of people living in coastal areas through permanent inundation and other SLR‐related hazards. Migration is one way for people to adapt to these coastal changes, but presents an enormous policy challenge given the number of people affected. Knowledge about the relationship between SLR‐related hazards and migration is therefore important to allow for anticipatory policymaking. In recent years, an increasing number of empirical studies have investigated, using survey or census data, how SLR‐related hazards including flooding, salinization, and erosion together with non‐environmental factors influence migration behavior. In this article, we provide a systematic literature review of this empirical work. Our review findings indicate that flooding is *not* necessarily associated with increased migration. Severe flood events even tend to decrease long‐term migration in developing countries, although more research is needed to better understand the underpinnings of this finding. Salinization and erosion do generally lead to migration, but the number of studies is sparse. Several non‐environmental factors including wealth and place attachment influence migration alongside SLR‐related hazards. Based on the review, we propose a research agenda by outlining knowledge gaps and promising avenues for future research on this topic. Promising research avenues include using behavioral experiments to investigate migration behavior under future SLR scenarios, studying migration among other adaptation strategies, and complementing empirical research with dynamic migration modeling. We conclude that more empirical research on the SLR‐migration nexus is needed to properly understand and anticipate the complex dynamics of migration under SLR, and to design adequate policy responses.

This article is categorized under:

- Climate Economics < Aggregation Techniques for Impacts and Mitigation Costs
- Vulnerability and Adaptation to Climate Change < Learning from Cases and Analogies
- Assessing Impacts of Climate Change < Evaluating Future Impacts of Climate Change

## Graphical abstract

Sea‐level rise (SLR) threatens millions of people living in coastal areas. Migration is one way for people to adapt to SLR‐related hazards, with major implications for people and policy. A more comprehensive understanding of migration dynamics under SLR is therefore vital.

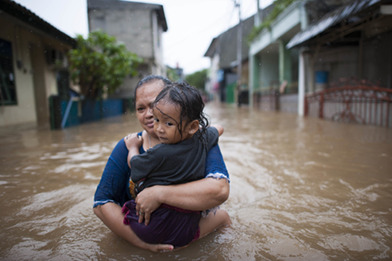

*Photo:* Flood event in Jakarta, Indonesia, 2017. *Photo source:* World Meteorological Organization on Flickr https://tinyurl.com/8azc3vnr

## 1 INTRODUCTION

According to the Intergovernmental Panel on Climate Change (IPCC) sixth assessment report, global mean sea levels have risen by around 0.2 m since 1900 (IPCC, 2021). Because of accelerating climatic change, the yearly rate of sea‐level rise has increased from 1.3 mm over the period 1901–1971 to 3.7 mm over the period 2006–2018, and is expected to increase further in the decades to come. Global mean sea‐level rise by 2100 relative to 1995–2014 is expected to be between 0.3 and 1 m depending on greenhouse gas emission scenarios. Higher levels approaching 2 m by 2100 and 5 m by 2150 cannot be ruled out because of deep uncertainty about ice sheet processes (IPCC, 2021). Sea‐level rise (SLR) posits an enormous threat to coastal populations, as it will lead to more frequent and intense flooding from storm surges, tidal extremes, waves, and backwater effects, and to coastal and riverbank erosion, water and soil salinization, and in some cases permanent inundation (Alam et al., 2016; Ketabchi et al., 2016; Nicholls & Cazenave, 2010; Vitousek et al., 2017). Next to climate‐induced SLR, other (non‐climatic) processes can further exacerbate these hazards, such as subsidence, dam construction, and industrial (dredging) activities (Dunn et al., 2018, 2019; Nicholls, Lincke, et al., 2021; Vasilopoulos et al., 2021). Economic development and population growth are expected to exacerbate vulnerability to SLR, although projections of these processes are uncertain as well (Jongman et al., 2012; Nicholls, Hanson, et al., 2021). These impacts affect coastal societies in numerous ways, such as by reducing agricultural production, threatening freshwater supplies, and damaging and destroying properties and critical infrastructure (Wrathall et al., 2019).

SLR‐related hazards could potentially displace tens of millions of people living in coastal areas by the end of this century (Hino et al., 2017; Lincke & Hinkel, 2021; Nicholls et al., 2011). Depending on population growth, more than one billion people could be populating the low‐elevation coastal zone and be vulnerable to SLR‐related hazards (Hauer, 2017; Neumann et al., 2015). However, other common and more restricted measures to assess “at‐risk” populations, including the 100‐year floodplain and areas inundated under SLR, suggest a much lower number (Hauer et al., 2020). Although of the SLR‐related hazards flooding threatens the greatest number of people, livelihoods of millions of people are also threatened by salinization and erosion impacts (Chen & Mueller, 2018; Hinkel et al., 2013; Nicholls et al., 2018). Policy measures focused on protection (i.e., armoring coasts to prevent SLR hazards) or accommodation (i.e., adaptation measures that facilitate living with SLR hazards) can mitigate the impacts of SLR. However, the high costs of these measures make it unlikely that they will be implemented along all threatened coasts (Hauer et al., 2020). Especially in developing countries and along low‐populated coastlines, protection is in many areas projected to be economically unfavorable under 21st century SLR (Lincke & Hinkel, 2018). When protection and accommodation are not feasible, retreat might be the only option left for adapting to SLR. Retreat can be centrally planned with the goal to relocate affected populations to safer areas, but as such government‐led resettlements are often expensive, complicated, and contested, its applicability seems limited to smaller populations highly vulnerable to SLR (Hauer et al., 2020; Wilmsen & Webber, 2015). The largest share of future SLR‐related migration will therefore likely be the result of people that autonomously decide to migrate in anticipation of, or in reaction to, SLR‐related hazards (McLeman, 2014). Such migration can be facilitated by government policy, for instance by prohibiting development in flood‐prone areas, not investing in flood protection, and property buyouts (Siders, 2013).

The locations from which, and how many, people will eventually migrate as a result of SLR and where they would migrate to is difficult to predict. Nevertheless, gaining a better understanding of future migration flows is of critical academic and policy relevance. It enables anticipatory policy planning to reduce the societal and economic costs of migration, can provide better insights into the welfare impacts of climate change, and allows for improved understanding and modeling of future coastal risk. Besides uncertainty about future SLR and policy responses, an important reason why forecasting migration under SLR is so difficult is the complexity of the migration decision‐making process, where next to SLR‐related hazards also demographic, cultural, economic, social, political, and individual/household‐level factors play a role (Black et al., 2011; Hauer et al., 2020; Wrathall et al., 2019). At present, economic and social motives instead of environmental concerns are often the main reason for people to migrate, also in vulnerable locations (Adger et al., 2021; Nicholls et al., 2020; Safra de Campos et al., 2020). Despite this complexity, we can already learn how SLR might influence migration from the growing empirical literature that investigates, using survey or census data, how SLR‐related hazards including flooding (Bohra‐Mishra et al., 2014; Call et al., 2017; Codjoe et al., 2017), erosion (Bernzen et al., 2019; Goldbach, 2017), and salinization (Bernzen et al., 2019; Chen & Mueller, 2018, 2019) impact migration or migration intentions. Although these hazards may not always be directly caused by SLR in the specific study settings, they reflect hazards that will be exacerbated by SLR, and are therefore relevant analogies.

This article presents the first systematic literature review that brings together these empirical studies. Our review focuses on those studies employing quantitative multivariate regression approaches because, despite the great value of qualitative research, this restriction facilitates the comparison of research findings and the isolation of SLR‐related hazards from other factors influencing the migration decision‐making process (Kaczan & Orgill‐Meyer, 2020). While previous review studies have been done on the impact of climate‐related events on human migration (see, e.g., Berlemann & Steinhardt, 2017; Black et al., 2013; Kaczan & Orgill‐Meyer, 2020; Klaiber, 2014), none of them centered around the impacts of SLR‐related hazards and as a result many relevant studies were excluded from these reviews. Moreover, the majority of the 15 studies included in this review were published since 2017, which indicates that reviews published before are outdated and miss many new studies. Kaczan and Orgill‐Meyer (2020) explicitly recognize the need for a systematic review of the impacts of SLR on migration, but yet do not consider these impacts themselves in their review of quantitative empirical studies on climate‐driven migration. Our study aims to fill this gap in the literature. Notwithstanding SLR is primarily a future affair, a “wait and see” approach is not an attractive option; instead the information available now can be utilized for making informed choices about how to anticipate and manage SLR‐induced migration.

To capitalize on the available information and to guide future research, this article provides two main outputs. First, embedding the findings from our literature review in a conceptual framework of human migration, adapted from Black et al. (2011) and Hauer et al. (2020), emerging patterns on the impact of SLR‐related hazards on human migration are discussed, as well as the role of non‐environmental factors. Second, this article proposes a research agenda for future empirical research on SLR and migration. Although this article takes stock of current knowledge, the evidence available is still sparse and confined to limited geographical contexts, which highlights the need for more empirical work on this topic (McLeman, 2014). We also argue that future empirical research should be more closely aligned to promising migration modeling approaches, such as agent‐based models (ABMs). ABMs can simulate the expected number of migrants under different SLR scenarios, where they come from and go to, and when they leave, while considering complex individual‐level and/or household‐level migration decision making processes (Kniveton et al., 2011; Speelman, 2015; Thober et al., 2018; Wrathall et al., 2019). This is relevant for policy making and goes beyond what can be achieved by empirical survey research alone (Williams et al., 2017; Wrathall et al., 2019). Key decisions that have to be made by developers of ABMs are the variables to include in the model and the decision‐making rules that agents (simulated individuals or households) follow. In the proposed research agenda, we discuss how empirical research can aid in making these decisions more well‐informed and improving modeling outcomes.

The remainder of this article is structured as follows. Section 2 discusses the conceptual framework. Section 3 explains the methodology used for the systematic literature review. Section 4 presents and discusses the review results. Section 5 proposes a research agenda for future empirical research on SLR and human migration. Section 6 concludes.

## 2 CONCEPTUAL FRAMEWORK

Migration can be defined as the permanent or semi‐permanent change of residence (Lee, 1966).^1^ Studying the impacts of environmental change on migration has become of increasing relevance due to climate change and widespread environmental degradation (McLeman & Gemenne, 2018). While initially studied predominantly by scholars from the broader natural sciences, the environmental migration literature is increasingly integrated with concepts, theories, and methodologies from the social sciences, and it is acknowledged that environmental factors are just one of many factors that influence the migration decision (Adger et al., 2018, 2021; Hunter et al., 2015; Nicholls et al., 2020; Safra de Campos et al., 2020).

**FIGURE 1:**
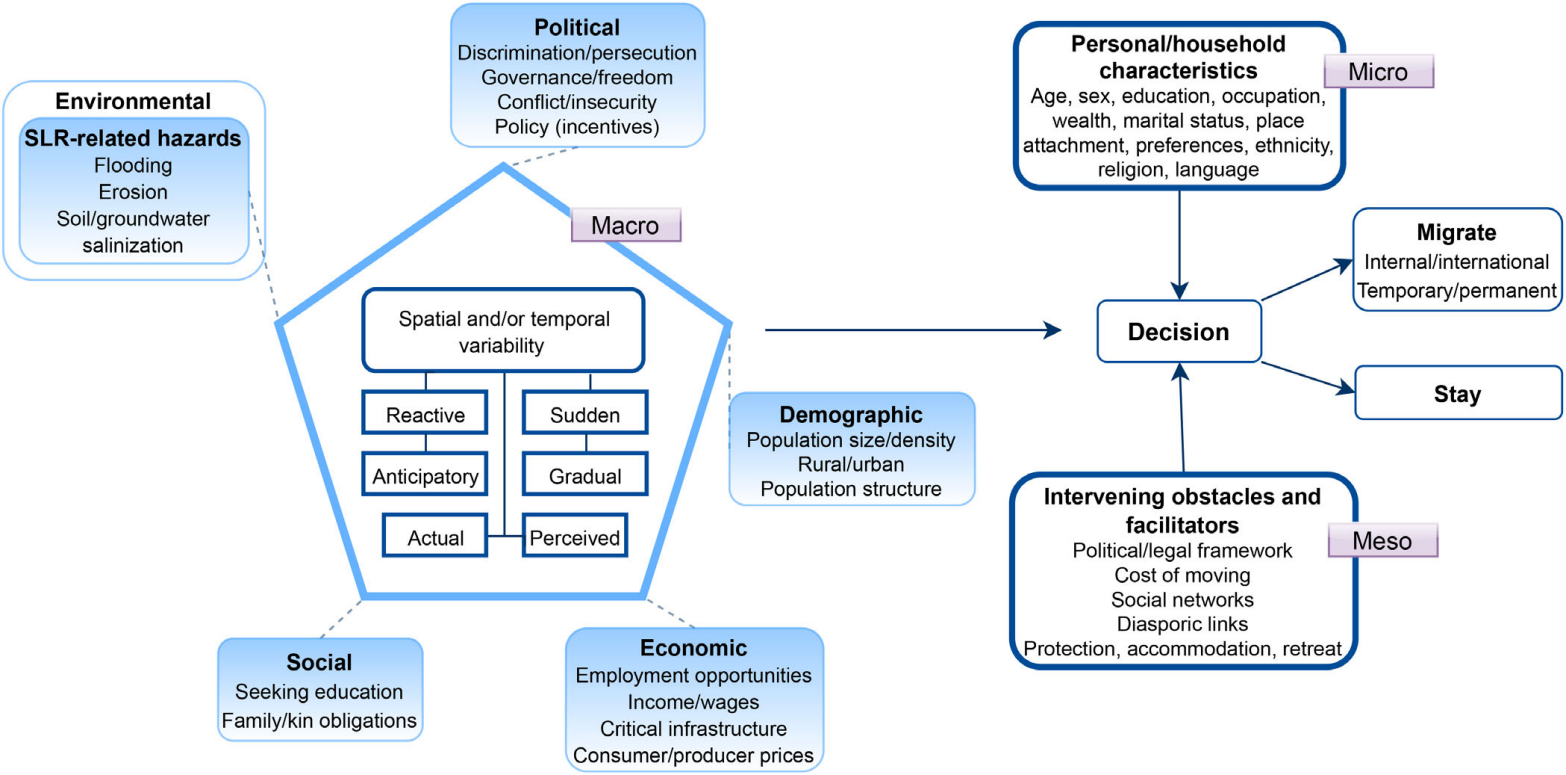
Migration in the context of SLR‐related hazards, modified from Black et al. (2011) and Hauer et al. (2020)

Figure 1 presents a conceptual framework of migration under SLR‐related hazards. This framework is modified from the original work of Black et al. (2011), and the later adaptation of Hauer et al. (2020) who applied the framework to SLR‐induced migration. The main benefit of this framework is that it recognizes the complexity of the migration decision‐making process and the associated drivers of migration (Hunter et al., 2015). In our adapted version, we take advantage of the richness of non‐environmental factors proposed by Black et al. (2011) and the application to SLR by Hauer et al. (2020). The framework depicts five macro drivers of migration in the context of SLR: economic, political, social, demographic, and SLR‐related hazards as a subset of environmental drivers. SLR‐related hazards in the conceptual framework and in the remainder of this article refer to hazards that are expected to be exacerbated by climate‐induced SLR, being flooding, salinization, and erosion. However, these hazards may also have other (non‐climatic) causes such as subsidence, dam construction, and industrial (dredging) activities (Dunn et al., 2018, 2019; Nicholls, Lincke, et al., 2021; Vasilopoulos et al., 2021). Environmental drivers of migration refer to the hazard people face (Black et al., 2011). Hence, studying people's migration response to (perceptions of) SLR‐related hazards, whether or not caused by climate‐induced SLR, provides us with important lessons on how people may respond to similar hazards under increased future SLR. SLR‐related hazards in the framework can influence migration directly or indirectly through the other four drivers. Within the pentagon, spatiotemporal variabilities in the migration response are shown. SLR‐related hazards can drive migration gradually (e.g., salinization) or suddenly (e.g., flooding from storm surges), and migration can be in anticipation or in reaction to SLR‐related hazards. Furthermore, both actual and perceived levels of risk can play a role. Whether drivers indeed translate to migration behavior is mediated by personal/household characteristics such as education, wealth, and preferences, and by intervening obstacles and facilitators such as the costs of migration, protection or retreat policies, and social networks. The framework reflects that both structural forces and human agency shape migration decisions, and it can be applied to different spatiotemporal types of migration. This conceptualization helps to classify and interpret the findings of our systematic literature review, and to identify knowledge gaps and fruitful avenues for future research.

## 3 METHODS

To synthesize current knowledge on the impact of SLR‐related hazards on migration, this study conducts a systematic review of the empirical literature. Appendix S1 provides a detailed description of the article inclusion criteria and the search and screening procedure of the review. To be included in the review, the following article inclusion criteria had to be met. First, the research should analyze migration intentions or actual migration as the dependent variable. Second, the research should study the effects of SLR‐related hazards, as an independent variable, on actual migration or migration intentions. Both studies looking at coastal and riverine flooding are included, as SLR through backwater effects can also increase the incidence and extent of upstream river flooding (Ikeuchi et al., 2015). Third, the study should utilize individual‐level or household‐level data. Fourth, data should be analyzed using multivariate regression analysis.

**FIGURE 2:**
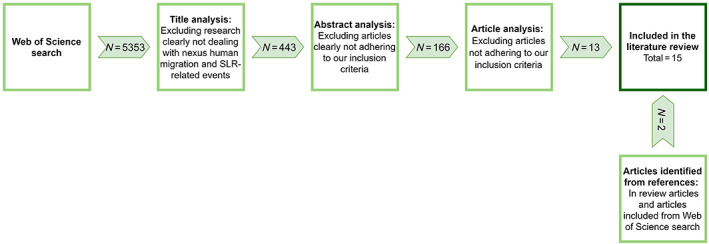
Selection process of articles included in the systematic literature review

We conducted the systematic literature search over the period November and December 2020 to identify peer‐reviewed scientific articles meeting all the inclusion criteria. A search term including keywords on SLR, migration, and empirical research methods was used and yielded 5353 articles. These articles were subsequently screened and filtered for eligibility following a three‐step procedure: title analysis, abstract and keyword analysis, and finally a thorough full‐text analysis. This resulted in 13 studies meeting all the inclusion criteria. We then screened the references of these 13 studies and the references of a selection of review articles dealing with migration and the environment, which yielded an additional two studies. The article selection process is summarized in Figure 2.

## 4 LESSONS FROM EMPIRICAL RESEARCH ON SLR‐RELATED HAZARDS AND HUMAN MIGRATION

### 4.1 Characteristics of articles included in the review

Table 1 provides an overview of the 15 articles included in the review, incorporating the following characteristics: (a) author(s) and year, (b) study area, (c) SLR‐related hazards studied, (d) how migration is measured, (e) type of migration studied in terms of migration destination and whether migration is temporary or permanent, and (f) survey or census data characteristics. The publication years of the articles included in this review lie between 2008 and 2020, with the majority of studies being published in the period 2017–2020. Geographically, the articles cover seven countries, being Bangladesh (five studies), the United States (four studies), Indonesia (two studies), Ghana (two studies), Australia (one study), Canada (one study), and Pakistan (one study). Most case studies comprise (at least partly) the coastal zone, except two. Regarding socio‐economic context, six studies are situated in developed countries, and nine studies in developing countries. In terms of SLR‐related hazards, the effect of flooding on migration is investigated in all included studies, and to a lesser extent salinization (three studies) and erosion (two studies) are also researched. For flood type, six studies focus on coastal flooding, three studies on riverine flooding, while six studies do not distinguish between flood types.

**TABLE 1:** Overview of studies included in the review

Articles	Study area	SLR‐related hazards	Migration measurement	Migration destination^a^	Temporary or permanent migration^a^	Data; collection year; *N*
Bernzen et al. (2019)	Coastal Bangladesh	Coastal/riverine flooding; erosion; salinization	Actual migration	Unspecified	Unspecified	Cross‐sectional; 2014; 1188 HH
Bohra‐Mishra et al. (2014)	Coastal Indonesia	Coastal/riverine flooding	Actual migration	Internal	Permanent	Panel; 1993–2007; 7185 HH
Boon (2014)	Coastal Australia	Riverine flooding	Migration intention	Unspecified	Unspecified	Cross‐sectional; 2010; 287 HH
Buchanan et al. (2019)	Coastal USA (New York)	Coastal flooding	Migration intention	Unspecified	Permanent	Cross‐sectional; 2016; 405 HH
Call et al. (2017)	Non‐coastal Bangladesh	Riverine flooding	Actual migration	Unspecified	Temporary	Panel; 1986–2003; ~200,000 IN
Chen and Mueller (2018)	Coastal Bangladesh	Coastal/riverine flooding; salinization	Actual migration	Internal; international	Permanent	Panel; 2003–2011; 550,473 HY
Chen and Mueller (2019)	Coastal and non‐coastal Bangladesh^b^	Coastal/riverine flooding; salinization	Actual migration	International	Unspecified^c^	Panel; 2005–2011; 1,288,982 HY
Codjoe et al. (2017)	Coastal Ghana	Coastal flooding	Migration intention	Unspecified	Unspecified	Cross‐sectional; 2012; 350 HH
Goldbach (2017)	Coastal Ghana and Indonesia	Coastal flooding; erosion	Actual migration	Short‐distance internal; long‐distance; combined	Unspecified	Cross‐sectional; 2015; 207 HH Ghana, 309 HH Indonesia
Gray and Mueller (2012)	Coastal and non‐coastal Bangladesh	Coastal/riverine flooding	Actual migration	Short‐distance internal; long‐distance; combined	Permanent	Panel; 1994–2010; 1680 HH
Haney (2019)	Non‐coastal Canada	Riverine flooding	Migration intention	Unspecified	Unspecified	Cross‐sectional; 2014; 407 HH
Mueller et al. (2014)	Coastal and non‐coastal Pakistan	Coastal/riverine flooding	Actual migration	Short‐distance internal; long‐distance; combined	Permanent	Panel; 1991–2012; 583 HH
Paxson and Rouse (2008)	Coastal USA (Louisiana)	Coastal flooding	Actual migration	Unspecified	Unspecified	Cross‐sectional; 2004–2007; 355 IN
Schwaller et al. (2020)	Coastal USA (North Carolina)	Coastal flooding	Migration intention	Unspecified	Unspecified	Cross‐sectional; 2017; 164 HH
Song and Peng (2017)	Coastal USA (Florida)	Coastal flooding; SLR generally	Migration intention	Unspecified	Unspecified	Cross‐sectional; 2014; 226 HH

Abbreviations: HH, households; HY, household‐years; IN, individuals.

^a^ This refers to whether the specification is made in the regressions analysis.

^b^ The impact of salinization is only assessed for coastal regions, the impact of flooding for both coastal and non‐coastal regions.

^c^ Individual moved away from the study area for at least 6 months, but return migration cannot be ruled out from the data.

The studies either use actual migration data (nine studies) or gauge migration intentions in survey questionnaires (six studies). Six out of the fifteen studies specify the migration destination (i.e., whether migration is characterized as short‐distance or long‐distance, internal, or international). Nine studies do not make this specification in their regression analyses, among which are all studies that investigate migration intentions. Six studies also specify the temporal dimension of migration (i.e., whether migration is temporary or permanent).^2^ Lastly, studies differ in terms of whether they use cross‐sectional data (nine) or panel data (six) for their regression analyses.

### 4.2 SLR‐related hazards and human migration

Table 2 presents the results of the 15 studies included in the review. The results are separately presented for the impacts of flooding, salinization, and erosion. Because of the heterogeneity in research approaches, the specific measurements of the independent variables are also described in the table. For each finding, the sign of the effect is shown, where a “+” indicates a positive impact on migration, and a “–” a negative impact. In addition, the significance level of the result is shown (*p* < 0.10, *p* < 0.05, or *p* < 0.01).^3^ Sometimes a range of significance levels is provided in case a study presents results of multiple model estimations. Non‐significant results are denoted by NS. Results are separated by migration destination, if applicable.

**TABLE 2:** Empirical evidence of relationship between SLR‐related hazards and human migration

Independent variable	Articles	Measurement of independent variable	Migration measurement; country	Migration destination			
				CM	SIM	LIM	INTM
*Flooding*							
Experience with flooding	Call et al. (2017)	Flood event in area in month of occurrence	Actual migration; Bangladesh	−(0.05)			
	Call et al. (2017)	Flood events in area in previous 12 or 24 months	Actual migration; Bangladesh	NS			
	Codjoe et al. (2017)	Self‐reported experience with coastal flooding	Migration intention; Ghana	NS			
	Goldbach (2017)	Flood risk in district based on 5‐year flood data	Actual migration; Indonesia	NS	NS	NS	
	Gray and Mueller (2012)	Household‐level exposure to flooding	Actual migration; Bangladesh	NS	NS	+(NS–0.10)	
	Haney (2019)	Self‐reported experience with home flooding	Migration intention; Canada	NS			
	Paxson and Rouse (2008)	Exposure of home to flooding	Actual migration; USA (Louisiana)	+(0.01)			
	Schwaller et al. (2020)	Self‐reported experience with home flooding	Migration intention; USA (North Carolina)	+(0.10)			
Severity or frequency of flooding	Bohra‐Mishra et al. (2014)	Province‐level impacts of flooding (number of deaths, injured people, and destroyed houses)	Actual migration; Indonesia		−(NS–0.05)		
	Boon (2014)	Self‐reported impacts of flooding (health problems, injuries, death of close relatives or friends)	Migration intention; Australia	+(0.01)			
	Buchanan et al. (2019)	Scenario of more frequent nuisance flooding (compared with less frequent major flooding)	Migration intention; USA (New York)	+(0.01)			
	Buchanan et al. (2019)	Scenario of extreme flooding (compared with less severe major flooding)	Migration intention; USA (New York)	NS			
	Chen and Mueller (2018)	Proportion of district flooded in past year	Actual migration; Bangladesh		NS		NS
	Chen and Mueller (2019)	Proportion of district flooded in past year	Actual migration; Bangladesh				−(0.10–0.01)
	Gray and Mueller (2012)	Percentage of households exposed to flooding in district	Actual migration; Bangladesh	NS	+(NS–0.01)^a^	−(NS–0.10)^a^	
	Mueller et al. (2014)	Flood intensity (province‐level number of deaths from flooding)	Actual migration; Pakistan	−(0.10–0.05)	−(0.10–0.01)	NS	
Economic losses from flooding	Bohra‐Mishra et al. (2014)	Province‐level financial losses from flooding	Actual migration; Indonesia		NS		
	Gray and Mueller (2012)	Household economic losses from flooding	Actual migration; Bangladesh	NS	NS	+(0.10)	
	Gray and Mueller (2012)	District economic losses from flooding	Actual migration; Bangladesh	NS	+(0.10)	NS	
Geographical risk of flooding	Bernzen et al. (2019)	Distance to river or coast	Actual migration; Bangladesh	−(0.10–0.01)			
	Call et al. (2017)	Distance to river	Actual migration; Bangladesh	+(0.01)			
	Goldbach (2017)	Distance to coast	Actual migration; Indonesia	−(0.10)	NS	NS	
	Goldbach (2017)	Distance to coast	Actual migration; Ghana	NS	NS	NS	
Flood risk perceptions	Haney (2019)	Belief that flooding becomes more frequent in the future	Migration intention; Canada	NS			
	Haney (2019)	Worry about future flooding affecting neighborhood	Migration intention; Canada	+(0.05–0.01)			
Other flood indicators	Goldbach (2017)	Feeling affected by flooding	Actual migration; Indonesia	NS	NS	NS	
	Goldbach (2017)	Feeling affected by flooding	Actual migration; Ghana	NS	NS	NS	
	Song and Peng (2017)	Self‐reported damage from flooding	Migration intention; USA (Florida)	NS			
Impact of flooding variables by gender	Chen and Mueller (2019)	Proportion of district flooded in past year	Actual migration; Bangladesh				Men −(0.10–0.01) Women NS
	Gray and Mueller (2012)	Household‐level exposure to flooding	Actual migration; Bangladesh	Men NS women NS			
	Gray and Mueller (2012)	Percentage of households exposed to flooding in district	Actual migration; Bangladesh	Men NS women + (NS–0.05)^a^			
	Mueller et al. (2014)	Flood intensity (province‐level number of deaths from flooding)	Actual migration; Pakistan	Men −(0.10) Women −(0.05)	Men −(0.10) women −(0.01)	Men NS women NS	
*Salinization*							
Experience with salinization	Bernzen et al. (2019)	Self‐reported freshwater availability problems from salinization	Actual migration; Bangladesh	NS			
	Chen and Mueller (2018)	Percentage of saline‐contaminated soil in district	Actual migration; Bangladesh		+(0.10)		−(0.05)^b^
	Chen and Mueller (2019)	Percentage of saline‐contaminated soil in district	Actual migration; Bangladesh				+(0.01)
Geographically at risk of salinization	Bernzen et al. (2019)	Living in shrimp farming dominated area	Actual migration; Bangladesh	+(0.05)			
Impact of salinization by gender	Chen and Mueller (2019)	Percentage of saline‐contaminated soil in district	Actual migration; Bangladesh				Men +(0.01) women +(0.01)
*Erosion*							
Experience with erosion	Bernzen et al. (2019)	Self‐reported loss of arable land, mainly due to riverbank erosion	Actual migration; Bangladesh	+(0.05–0.01)			
	Goldbach (2017)	Self‐reported experience with coastal erosion	Actual migration; Indonesia	NS	NS	NS	
	Goldbach (2017)	Self‐reported experience with coastal erosion	Actual migration; Ghana	NS	NS	NS	
	Goldbach (2017)	Physical coastal erosion risk in district	Actual migration; Ghana	NS	NS	NS	
*SLR in general*							
SLR perceptions	Song and Peng (2017)	Believe in SLR occurring and believe in SLR intensifying extreme weather events	Migration intention; USA (Florida)	+(0.05)			

*Note*: −/+ is the sign of the effect, significance level in parentheses, NS means effect is not significant. Results displayed in merged cells indicate that the measure encompasses multiple migration destinations.

Abbreviations: CM, migration destination combined or unspecified; INTM, international migration; LIM, long‐distance internal migration; SIM, short‐distance internal migration.

^a^ Only moderate flooding intensity has a significant impact (i.e., when 5%–20% of households in district are exposed to flooding), more severe flooding has not (i.e., >20% of households are exposed).

^b^ For international migration within South Asia, however, salinization does have a positive effect on migration (*p* < 0.05).

#### 4.2.1 Flooding

All studies included in this review studied the effect of flooding on migration. Different types of flood indicators are studied: flood experience, flood severity or frequency, economic damages from flooding, geographical indicators of flood risk, and flood risk perceptions. Of the eight flood experience indicators five are reported as insignificant, two as having a positive impact on migration, and one as having a negative impact on migration. Positive impacts of flood experience on migration are found in US studies by Paxson and Rouse (2008) and Schwaller et al. (2020), for experience with home flooding specifically. Home flooding can cause serious economic damage and experiencing it first‐hand can strongly increase risk perceptions of flooding, as explained by the availability heuristic (Botzen et al., 2021), which can promote migration behavior. However, for the other studies we find insignificant or even negative impacts of flood experience on migration. This discrepancy might be due to the fact that these studies were conducted in developing countries (except for Haney, 2019, who finds a positive, albeit insignificant, effect on migration intentions in a Canadian study). People in developing countries are often more dependent on coastal and riverine resources for their livelihoods, and also face higher financial constraints, which may limit their ability to migrate. These financial constraints can be further exacerbated by flood events. This is exemplified by Call et al. (2017) who find that a river flood event in the month of occurrence significantly reduces temporary migration in Bangladesh, with the odds of migration being 17% lower in a month of flooding compared with a month without flooding. Besides financial constraints, the flood type studied could also explain the divergence in results. River flooding, as opposed to coastal flooding, provides freshwater that can boost agricultural productivity and may reduce migration in agricultural households (Banerjee, 2010; Chen & Mueller, 2019). As will be explained in Section 5 in greater detail, more research evidence is needed to validate and clarify this possible multidirectional relationship between flooding and migration.

A similar discrepancy between developed and developing country case studies can be observed when we look at indicators of flood severity or frequency. Studies focusing on Bangladesh, Indonesia, and Pakistan find that more severe floods in terms of deaths, destroyed houses, or exposed households significantly decrease long‐term migration (Bohra‐Mishra et al., 2014; Chen & Mueller, 2019; Mueller et al., 2014). Contrastingly, studies in Australia and New York find that more severe or frequent flooding increases intentions to migrate (Boon, 2014; Buchanan et al., 2019). However, the measurement of migration could also play a role here. Namely, the studies that focused on developing countries looked at actual migration flows, while those focusing on developed countries studied migration intentions. Flood type (coastal or riverine), which in some of these studies is unspecified, could also moderate the effects found. Nonetheless, this does not undermine the finding that severe flooding tends to restrict long‐term migration in developing country contexts. Although severe flooding can generate large temporary population displacements in developing countries (IDMC, 2017), displacement may often not result in permanent migration because people prefer to return to their place of residence for social or economic reasons, or because severe floods deplete household resources to finance migration (McLeman, 2014). Looking at less severe “moderate” flood events, Gray and Mueller (2012) find that this increases short‐distance migration and decreases long‐distance migration in Bangladesh. In contrast to severe flood events, moderate flood events might leave enough household resources to allow for short‐distance migration (Kaczan & Orgill‐Meyer, 2020). In light of the discussion on the possibly important role of financial constraints in developing countries, it is surprising that Bohra‐Mishra et al. (2014) and Gray and Mueller (2012) find that indicators of economic losses from flooding are insignificantly or weakly positively associated with migration. However, both studies use measures of absolute economic losses. Absolute economic losses from flooding may be higher for richer households, for whom migration may still be affordable.

Risk measured in terms of the distance people live from rivers and coasts is significantly associated with migration in three out of four studies looking at this geographical indicator. Goldbach (2017) and Bernzen et al. (2019) find in, respectively, Bangladesh and Indonesian case studies that people living closer to a coast or river are more likely to migrate. In contrast, Call et al. (2017) in a Bangladesh case study find that people living closer to a river are less likely to migrate. The choice of living close to coastal or riverine waterbodies reflects a trade‐off between greater economic and livelihood opportunities and increased flooding and other SLR‐related hazards. However, riverine flooding also comes with benefits by irrigating the land with freshwater (Banerjee, 2010). These benefits of riverine flooding may explain the positive effect found by Call et al. (2017), who focus exclusively on the distance to rivers.

Two studies in our review investigate the relationship between migration and self‐reported risk perception. Haney (2019) finds that people in the Canadian inland city of Calgary who believe that flooding will become more frequent in the future do not report higher migration intentions, while people who worry about future flooding affecting their neighborhood are significantly more likely to intend to migrate. In fact, worried individuals report a 67% lower likelihood of staying in their neighborhood in the coming year, and a 50% lower likelihood of staying for the coming 5 years, compared with non‐worried individuals. Song and Peng (2017) in a US study find that both a belief that SLR is occurring and a belief that SLR will intensify extreme weather events is significantly positively associated with migration intentions. Both findings illustrate the important role that risk perceptions can play alongside actual risk, as is also pointed out in Figure 1. The influence of risk perceptions is particularly relevant for SLR, which main impacts lie in the future and can be gauged by self‐reported risk perceptions.

Chen and Mueller (2019), Gray and Mueller (2012), and Mueller et al. (2014) compare the migration impacts of flooding by gender. Chen and Mueller (2019) find that in Bangladesh more severe flooding decreases migration among men, but not among women. Gray and Mueller (2012) in another Bangladesh study find that indicators of flood severity are negatively associated with migration among men, albeit insignificantly, while they tend to be positively associated with migration among women, and significantly so for moderate flood severity. Mueller et al. (2014) find that flooding in Pakistan significantly deters short‐distance migration for both males and females but find no significant effects for long‐distance migration. The finding of the two Bangladesh studies that flooding has more negative effects on migration for men than for women is somewhat unexpected, given that men generally have more migration decision‐making power than women in developing countries, and men are also more likely than women to move for economic purposes (Kaczan & Orgill‐Meyer, 2020). It could be that migration decreases more among men than among women when communities have to be rebuild after a severe flood event. In addition, migration for marriage, one of the most important reasons for migration among Bangladeshi women, might be little affected or even increase after flooding as it can be seen as a risk diversification strategy for resource‐constrained households (Rosenzweig & Stark, 1989).

Whether studies focus on migration intention or on actual migration also seems to matter for the study outcomes. In studies that investigate migration intention, the effects of the flooding indicators are either insignificant or positive. Studies that investigate actual migration find that flooding can both decrease and increase migration, depending on the type of flood indicator and study context. Besides variations in study context, the intention–behavior gap could be a reason for the observed differences, as intentions do not always lead to action (Echegaray & Hansstein, 2017). Although people might intend to leave because of flooding, they may change their minds over time or find themselves physically, financially, or in any other way constrained in their migration decision. These diverging findings could also disclose that flooding impacts migration over longer time horizons than shortly after flooding events; flooding can influence migration intentions or migration decisions, but this might not yet be revealed through actual migration behavior. Reduced (financial) capability to migrate shortly after flood events can be one reason for this (Kaczan & Orgill‐Meyer, 2020).

#### 4.2.2 Salinization and erosion

In contrast to flooding, much less research has been done on the impact of salinization on migration, and the studies included in this review are confined to Bangladesh. However, the results that are available provide a more consistent picture of the relationship between migration and salinization than is the case for flooding. In most cases, experience with or being geographically at risk of salinization increases migration (Bernzen et al., 2019; Chen & Mueller, 2018, 2019). Although Chen and Mueller (2018) find that salinization deters international migration overall, salinization still has a significant positive influence on international migration to nearby countries in South Asia. Perhaps negative economic effects as a result of salinization can obstruct long‐distance international moves. Chen and Mueller (2019) find that a one standard deviation increase in soil salinity increases migration from coastal Bangladesh to India and other countries in the region by a factor of 18, leading to an expected yearly increase in outmigration of 17,874 people to India alone (based on 2011 data). Chen and Mueller (2019) also examine whether the impact of salinization differs by gender, but find that salinization increases migration among both men and women. Salinization in Bangladesh greatly reduces yields of rice and other important crops, and as a result threatens agricultural production and people's livelihoods (Clarke et al., 2015; Hussain et al., 2018). There is now a widespread conversion from paddy cultivation to aquaculture in saline‐affected areas in the country, increasing rural unemployment and poverty, and stimulating out‐migration (Lázár et al., 2020; Nicholls et al., 2018). This can all explain the strong positive effect of salinization on migration found in our review. In addition, while sudden financial losses from extreme flooding events can deter people's capability to migrate, salinization is more of a gradual process where people have the time to plan migration and collect the necessary resources (Chen & Mueller, 2019; Kaczan & Orgill‐Meyer, 2020). These findings on the impact of salinization in coastal Bangladesh correspond to Li et al. (2014) who in a case study in inland China find that individuals who experienced problematic salinization as a result of excessive groundwater extraction were much more inclined to migrate than individuals who did not experience these difficulties.

Only two studies in this review looked at how erosion in coastal areas influences migration. Goldbach (2017) finds that coastal erosion does not affect migration in both Ghana and Indonesia. A reason for this insignificant effect could be that coastal erosion only impacts migration for people living directly adjacent to the coast, while in the study of Goldbach (2017) most respondents lived a few kilometers away from the shoreline. In another study, Bernzen et al. (2019) focused on loss of arable land in Bangladesh and find that this does affect migration. Over 93% of respondents reported that riverbank erosion was the reason for the arable land loss. Future SLR, by intensifying the hydrological cycle and river flooding, is expected to enhance the loss of arable land as a result of riverbank erosion (Alam et al., 2016). Loss of arable land impedes agricultural and housing opportunities and, consequently, may force people to migrate (Islam & Rashid, 2011). In fact, in Bernzen et al. (2019) those who experienced arable land loss were 2.5 times more likely to migrate than those who did not. In the scientific and in the public discourse flooding as a result of SLR captures most attention (Nicholls, 2011), but the previous findings illustrate that salinization and erosion and their relationship with migration should be seriously considered as well.

### 4.3 Other factors that influence migration

Table A1 in the Appendix presents an overview of non‐environmental factors and their effects on migration, as obtained from the results of the 15 studies included in our review. These factors cover many, but certainly not all, elements shown in the conceptual framework in Figure 1. Especially information on the impact of macrolevel factors, such as government policies, is still lacking. We only discuss factors that have been investigated in at least two study settings, so that the patterns found are more reliable.

We identify several factors that play a role in explaining migration behavior next to SLR‐related hazards. First, individuals with higher income/wealth are in most cases more likely to migrate than poorer individuals. Although the poor and marginalized are often hit hardest by natural disasters, a lack of economic resources can inhibit their capability to migrate, which can be further exacerbated by natural hazard events (Black et al., 2011; Kaczan & Orgill‐Meyer, 2020). However, Gray and Mueller (2012) find that coming from a richer household significantly increases migration among men but not among women in Bangladesh, reflecting potential gender inequalities in the capability to capitalize on higher household resources. Second, homeowners are less likely to migrate. Homeownership indicates a substantial economic investment that ties people to their place of residence, and may also indicate place attachment (Haney, 2019). Third, and related to the previous point, place attachment is associated with a lower intention to migrate, which is a commonly held view in the literature (Adams & Kay, 2019; Swapan & Sadeque, 2021). Fourth, two US studies find that black people are more likely to migrate than people of other ethnicities (Paxson & Rouse, 2008; Song & Peng, 2017). This could reflect that black communities in the United States are disproportionally located in areas most vulnerable to climate change (Shepard & Corbin‐Mark, 2009), as well as that black people are more likely to be affected by the phenomenon of climate gentrification (Shokry et al., 2020). This raises an important issue, and policy should prevent that already existing inequalities will be aggravated by SLR. Fifth, people with social networks outside of their place of residence are more inclined to migrate. External social networks reduce information costs and make it easier for migrants to integrate into their new destination, and are central in network theories of migration (Haug, 2008; McLeman, 2014; Ryan, 2011). Finally, individual preferences including risk aversion and impatience have been found to lower the likelihood of migration. This can be explained by the fact that migration involves a high level of uncertainty, and it might take time before one can reap the benefits of a migration investment (Goldbach, 2017). We observe no clear influence of age, education, or gender. Although younger, male, and higher educated individuals are often depicted as being more likely to take part in environmental migration, especially internationally (Kaczan & Orgill‐Meyer, 2020; Obokata et al., 2014), this general pattern is not observed for the studies included in this review.

## 5 A RESEARCH AGENDA FOR FUTURE EMPIRICAL RESEARCH ON SLR AND MIGRATION

The previous section presented the results of a systematic literature review concerning empirical research on the impact of SLR‐related hazards on human migration. Based on these results, the following section proposes a research agenda by discussing the main gaps that still exist in the literature and fruitful avenues for future research. With this research agenda, we aim to help bring the science forward and promote research that provides policymakers and other stakeholders with the information they need to anticipate and manage SLR‐related migration. The research agenda discusses seven main themes, identifying research avenues for: (a) empirical research for varying geographical contexts and SLR risks, (b) studying a broader set of explanatory variables, (c) utilizing behavioral experiments and risk perception data, (d) differentiating impacts of SLR for subpopulations, (r) defining migration specifics, (f) researching migration together with other adaptation options, and (g) making empirical research complementary to dynamic migration modeling. Consistent with our systematic literature review, this research agenda focuses on quantitative research. Although not discussed in detail here, we acknowledge the importance of qualitative research. Qualitative research allows for a more in‐depth and nuanced understanding of migration motives, and is of high value in identifying the role of non‐environmental factors like gender and culture, and how these factors interact with environmental change such as SLR (Gioli & Milan, 2018; McLeman & Gemenne, 2018; Zickgraf, 2019). In addition, qualitative work offers important insights and experiences from marginalized and indigenous people (Albert et al., 2018; Farbotko, 2018), whose specific views are not reflected in our review study.

1. *Empirical research for varying geographical contexts and SLR risks*. Despite the wealth of knowledge gained from the 15 articles included in the systematic literature review, our understanding of the issue is still limited. One major limitation is the geographical scope of the current literature. Only seven country contexts are included in our review. Quantitative empirical research is lacking in some of the countries expected to be among the hardest hit by SLR, including China, India, Vietnam, and the African countries of Egypt and Nigeria (Hauer et al., 2020). Studies are also lacking in several developed countries with considerable population numbers in the low‐elevation coastal zone, such as the Netherlands and the United Kingdom (Neumann et al., 2015). Migration is generally a less pressing issue in these contexts because coastal protection is put in place and planned for, and is often economically feasible under future SLR scenarios (Lincke & Hinkel, 2018; McEvoy et al., 2021). For instance, in the Netherlands coastal protection currently insulates the population from SLR changes and flood risk perceptions are generally very low (Kellens et al., 2013; Mol et al., 2020). Such case study locations would only become of substantial relevance for SLR migration research would protection fail or risk perceptions increase for other reasons like public awareness programs. Another limitation of present research is the uneven distribution of SLR‐related hazards that are studied, with erosion and salinization being understudied compared with flooding. This balance should be improved as worldwide many countries already experience the impacts of salinization as well as coastal and riverbank erosion, which will increase under future SLR (Das et al., 2014; Mentaschi et al., 2018; Smajgl et al., 2015). Future studies focusing on flooding should differentiate between the type of flood event, as the impacts of coastal compared with riverine flooding may be different, sometimes interacting in coastal zones, but are often not accounted for in current studies. These studies could also help to validate and clarify the multidirectional relationship between flooding and migration suggested from the findings of our review, disentangling the role of possibly decisive factors such as flood type and financial constraints. Finally, when studying the impacts of SLR‐related hazards on migration, studies should consider not only the role of climate‐induced SLR but also that of other (non‐climatic) factors driving these hazards, including subsidence (Nicholls, Lincke, et al., 2021).
2. *Broadening of the set of explanatory variables*. The conceptual framework portrayed in Figure 1 theorizes the factors that can be important in explaining migration in the context of SLR. However, many of these factors are not or barely analyzed in the empirical literature. Macrolevel drivers, including economic, social, demographic, and political drivers, are especially understudied. Of particular importance is the impact of government policies on migration decisions, such as adaptation or disaster insurance policies (Wrathall et al., 2019). As these are important tools that governments could use to steer migration, research is needed to provide insights into their effectiveness. Besides using data from available datasets, the effects of macrolevel variables can also be tested in behavioral field experiments or by collecting survey data on people's perceptions, which could be necessary if the geographical scope of the study does not include variation in macrolevel context. Future research could also focus more on the influence of intervening obstacles or facilitators of migration. This includes the costs of migration, which is considered to be of high importance in the migration decision. However, in the literature its impact is often only gauged indirectly by looking at household wealth measures (Kaczan & Orgill‐Meyer, 2020). The effect of pull, compared with push, factors also deserves more scholarly attention.
3. *Utilization of behavioral experiments and risk perception data*. Many of the studies included in the literature review presented in this article have analyzed how SLR‐related hazards (i.e., flooding, salinization, and erosion) in the past have influenced migration. Although learning from the past gives us important insights, the dynamics of these hazards under future SLR will be markedly different and so will be their impact on migration. When flooding becomes more frequent and severe, and erosion and salinization increases, migration thresholds may be reached that have not been reached in the past. Currently, knowledge about migration thresholds for people under climate change and SLR is increasing but still limited, and more research is needed on what levels and manifestations of SLR trigger (inevitable) migration (Hauer et al., 2020; McLeman, 2018). To circumvent the drawbacks of using data on past migration, behavioral experiments and risk perception studies can be utilized. However, these studies are currently underrepresented in the literature. Research on risk perceptions can help us better understand how SLR already influences migration also beyond hazard exposure or impacts, for instance by studying if people who understand or are worried about SLR are more likely to migrate from vulnerable areas. This also allows for the incorporation of local knowledge in addition to scientific measures of SLR risk (McMichael et al., 2021). This can also improve our understanding of anticipatory migration patterns, instead of reactive migration where most studies so far have focused on. Behavioral experiments, on the other hand, can be utilized to identify when thresholds are reached and people start to migrate. This can be done by presenting respondents with migration and adaptation choices under realistic future SLR scenarios for permanent inundation, flooding, salinization, and/or erosion and analyzing the choices they make. Choice experiments are an example of behavioral experiments that could be used for such purposes (Dachary‐Bernard et al., 2019; Train, 2009). Results of behavioral experiments could provide pivotal information on when thresholds are reached for people to migrate, and consequently on when to anticipate migration under different SLR scenarios. Longitudinal, in addition to cross‐sectional, studies could help identify if results differ before and after experience with SLR‐related events such as flooding, or whether people actually migrate when they experience the SLR‐related hazards they were presented with in hypothetical experiments (Bubeck et al., 2012).
4. *Differentiation of the impacts of SLR for subpopulations*. The impacts of SLR on migration are not expected to be uniform across populations. For instance, vulnerable population groups like women, the elderly, the poor, and minorities, tend to be less capable of acting on climate risks, especially in developing countries (Gioli & Milan, 2018; Otto et al., 2017; Rahman, 2013). However, this heterogeneity is not properly accounted for in the analytical strategies of most of the quantitative empirical literature, which is characterized by the use of linear regressions on the full sample. Better accounting for population heterogeneity is imperative, and prevents oversimplification of the environment‐migration relationship. To do this, future studies could estimate separate regressions for different population groups, such as is done by Mueller et al. (2014) for gender, or include interaction effects between SLR and socio‐demographic indicators (e.g., interacting soil salinity with income). It should be taken into account that for reliable results these analyses might require large sample sizes.
5. *Defining migration specifics*. Migration specifics, including when, where and for how long people migrate, are often not well defined, whereas this is pivotal in properly understanding migration dynamics and their causal factors. For instance, only 6 out of the 15 studies in our review specify the migration destination or whether migration is temporary or permanent. Especially migration intention research tends to ignore migration specifics, where often broad dichotomous questions are analyzed on whether respondents plan to migrate in the future, but without considering its particularities. Migration intention research can help identify migration thresholds under future SLR, but loses much of its value when we do not know what this migration will look like. Another important aspect in this domain is that of migrant agency, which is about the agency or freedom an individual/household has in the migration decision‐making process (Bakewell, 2010; McLeman, 2014). Migrant agency represents a continuum with fully voluntary and involuntary (forced) migration as the two extremes. The degree of migrant agency in the context of SLR risk may vary (McLeman, 2014), and is important to examine in future research.
6. *Researching migration as part of a broader array of adaptation options*. Most studies research migration as the only outcome, whereas people can adapt to SLR in many other ways than migration, such as by flood proofing their houses or by cultivating saline‐tolerant crops (Jamero et al., 2017; McLeman, 2014). Therefore, one of the greatest challenges for future research is to break the silos of the migration and mainstream adaptation literature, and to bundle strengths to explain how people respond to SLR risk. Compared with the migration literature, the literature on in‐situ adaptation to SLR‐related hazards is much larger and further developed, with most attention being focused on flood adaptation (Bubeck et al., 2012; Koerth et al., 2017; Paik et al., 2020). Combining both strands of literature could provide important insights into the circumstances under which people will implement private adaptation measures to protect themselves against SLR, when people will decide to migrate, how these relate to each other, and which response will be more likely under future SLR scenarios. A choice experiment analysis in the United States by Buchanan et al. (2019) where respondents under different flood scenarios choose whether to take up insurance, elevate their homes, or migrate, provides a good example of such an integrative approach.
7. *Making empirical research complementary to migration modeling research*. It is of high policy relevance to obtain reliable forecasts on the key dimensions of human migration under future SLR: how many people will migrate, where they will migrate to, the timing of their migration, and their socio‐economic situation before and after migration (McLeman & Gemenne, 2018; Wrathall et al., 2019). Dynamic migration modeling approaches can be employed to obtain such forecasts, and examples include gravity models (Afifi & Warner, 2008), radiation models (Davis et al., 2018), machine learning models (Robinson et al., 2020), and agent‐based models (Bell et al., 2021). Although all these model types have their merits, in the remainder our focus will be on agent‐based models (ABMs) because of their unique capability to model complex individual decision‐making processes while incorporating socio‐ecological interactions at the individual/household level (Klabunde & Willekens, 2016). ABMs can take into account heterogeneity in the characteristics of individuals/households, and aggregate micro‐level results to population‐level migration outcomes (Klabunde & Willekens, 2016; Kniveton et al., 2011; Thober et al., 2018; Wrathall et al., 2019). The use of ABMs has become increasingly popular among migration scholars, and a few ABM studies have simulated migration under future SLR (e.g., Bell et al., 2021; Hassani‐Mahmooei & Parris, 2012). Empirical studies, like the studies included in our review, have an important role to play in developing realistic ABMs, and as a result in making their outcomes useful for policymakers (Gray et al., 2017; Schlüter et al., 2017; Williams et al., 2017). We highlight two ways in which empirical research can contribute, both reflecting key decisions that have to be made by ABM modelers. First, ABM modelers select and parametrize a set of variables that influence migration behavior in the model. Quantitative survey‐based research can facilitate this by providing insights into the main factors that influence migration and their effect sizes. Second, ABM modelers decide about the behavioral decision‐rules that agents follow (i.e., how individuals/households make decisions in the model), which are preferably guided by behavioral decision theory (Groeneveld et al., 2017). The choice and formalization of a theory can determine to a large extent the outcomes of the model, and it is therefore important that a theory is chosen that is most appropriate for the specific research context (Klabunde & Willekens, 2016; Schlüter et al., 2017; Wens et al., 2020). Some of the most commonly used behavioral theories in social‐ecological ABMs include economic theories such as Subjective Expected Utility Theory and Prospect Theory and psycho‐sociological theories such as the Theory of Planned Behavior and Protection Motivation Theory (Aerts, 2020; Schlüter et al., 2017). Empirical survey‐based research can aid in the choice and the subsequent calibration of a behavioral theory. For instance, our review of the empirical literature illustrates that economic wealth can play an important role in whether someone migrates after SLR disasters. This seems to indicate that people's (economic) capability to migrate is an important factor in the migration decision, which is one of the key elements in the Theory of Planned Behavior and Protection Motivation Theory, and can be incorporated in economic decision models as budget constraints. Applying behavioral theories that approach the true complexity of human behavior would be an important step in designing realistic ABMs, as at present still much of the ABM literature is not firmly grounded in behavioral theory or assumes rational agent behavior (Gray et al., 2017; Groeneveld et al., 2017; Schlüter et al., 2017). There is also potential in integrating the migration and adaptation literature in ABM modeling, so that future pathways of both SLR‐induced adaptation and migration can be simulated. Indeed, much ABM development is already going on in the flood adaptation literature (Haer et al., 2019; Zhuo & Han, 2020), and we encourage exploring cross‐fertilizations between both strands of research.

## 6 CONCLUSION

SLR presents a substantial threat to hundreds of millions of people living in coastal areas. Migration is one way for people to adapt, and may increasingly become the preferred option when SLR impacts intensify in the future. Knowledge about migration dynamics under SLR is important to allow for anticipatory policymaking and to better understand the welfare impacts of climate change. In this article, we present the results of a systematic review of the empirical literature and bring together existing knowledge on the relationship between migration behavior and SLR‐related hazards including flooding, salinization, and erosion. This review adds to existing reviews on climate change and human migration by focusing specifically on SLR‐related hazards, which is valuable given the growth in empirical studies on this topic in recent years. Findings from our review indicate no clear association between flooding and higher levels of migration. Severe flooding is often even associated with decreased levels of long‐term migration in developing countries, although the underpinnings of this finding remain unclear from present research. Salinization and erosion have been investigated in fewer studies, in which they tend to be associated with increased levels of migration. Important factors that influence migration alongside SLR‐related hazards include income, homeownership, place attachment, race, social networks, and risk attitudes.

Despite an increase in scholarly attention in recent years, the number of studies on SLR‐related hazards and migration is still limited and there are promising ways to improve upon existing findings. Therefore, we also presented a research agenda outlining knowledge gaps in the literature and fruitful avenues for future research. We highlight that dynamics of SLR‐related hazards such as flooding will be markedly different under future SLR than they were in the past. As a result, we encourage research to investigate how people will likely respond to future SLR scenarios, for instance by utilizing behavioral experiments. We also encourage scholars to clearly define migration specifics, expand the geographic contexts and explanatory variables that are investigated, differentiate impacts of SLR for subpopulation groups, and study migration as part of a broader array of adaptation strategies that people can choose. Finally, we discuss how empirical research can aid agent‐based modeling, which is a promising approach to forecast migration flows under future SLR scenarios. What is clear is that more empirical research is needed if we want to properly understand the complex dynamics of migration under SLR and anticipate it in a timely manner. We hope that our work can give directions for such endeavors.

## CONFLICT OF INTEREST

The authors have declared no conflicts of interest for this article.

## AUTHOR CONTRIBUTIONS

**Sem Duijndam:** Conceptualization (lead); data curation (lead); formal analysis (lead); investigation (lead); methodology (lead); validation (equal); visualization (lead); writing – original draft (lead); writing – review and editing (lead). **Wouter Botzen:** Conceptualization (supporting); data curation (supporting); formal analysis (supporting); investigation (supporting); methodology (supporting); validation (equal); visualization (supporting); writing – original draft (supporting); writing – review and editing (supporting). **Liselotte Hagedoorn:** Conceptualization (supporting); data curation (supporting); formal analysis (supporting); investigation (supporting); methodology (supporting); validation (equal); visualization (supporting); writing – original draft (supporting); writing – review and editing (supporting). **Jeroen Aerts:** Conceptualization (supporting); data curation (supporting); formal analysis (supporting); funding acquisition (lead); investigation (supporting); methodology (supporting); project administration (lead); validation (equal); visualization (supporting); writing – original draft (supporting); writing – review and editing (supporting).

## RELATED WIREs ARTICLES

A mobilities perspective on migration in the context of environmental change

Spatial and temporal ways of knowing sea level rise: Bringing together multiple perspectives

Integrating new sea‐level scenarios into coastal risk and adaptation assessments: An ongoing process

## Supporting information


**Appendix S1** Supporting Information

## Data Availability

Data sharing is not applicable to this article as no new data were created or analyzed in this study.

## Endnotes

^1^
Migration as a result of SLR‐related hazards is characterized as environmental migration. We define environmental migrants in this article following the definition of Kaczan and Orgill‐Meyer (2020) and the International Organization for Migration (2009): “Environmentally induced migrants are persons or groups of persons who, for compelling reasons of sudden or progressive changes in the environment that adversely affect their lives or living conditions, are obliged to leave their habitual homes, or choose to do so, either temporarily or permanently, and who move either within their country or abroad” (Kaczan & Orgill‐Meyer, 2020, p. 282).

^2^
Most migration intention studies do not specify whether migration is temporary or permanent, and only investigate whether respondents intend to “migrate” from their place of residence. Some studies focusing on actual migration flows do also not make this specification.

^3^
We do not report the size of the regression coefficients. Because studies did not report standardized regression coefficients, the size of the regression coefficients depends on the scales and units of the independent variables used in the specific studies, which impedes a meaningful comparison across studies. We therefore recommend future research to report standardized regression coefficients.

## Other variables and their influence on human migration in the context of SLR

**TABLE A1:** Influence of other variables on human migration in the context of SLR

Independent variable	Articles	Migration measurement; country	Migration destination			
			CM	SIM	LIM	INTM
*Personal/household characteristics*						
Age	Call et al. (2017)	Actual migration; Bangladesh	−(0.01)			
	Codjoe et al. (2017)	Migration intention; Ghana	NS			
	Goldbach (2017)^a^	Actual migration; Indonesia	Age + (0.01), age ^ 2 − (0.01)			
	Goldbach (2017)	Actual migration; Ghana	−(NS–0.01)			
	Gray and Mueller (2012)^b^	Actual migration; Bangladesh	Total +(0.05–0.01) Men +(0.01) Women +(NS–0.01)	+(NS–0.01)	+(0.01)	
	Haney (2019)	Migration intention; Canada	Age −(0.01), age ^ 2 + (0.05)			
	Mueller et al. (2014)^b^	Actual migration; Pakistan	Men + (0.01) Women NS	Men +(0.01) Women NS	Men +(0.01) Women NS	
Age of household head	Bohra‐Mishra et al. (2014)	Actual migration; Indonesia		−(0.01)		
	Mueller et al. (2014)	Actual migration; Pakistan	Men NS Women NS	Men NS Women NS	Men NS Women NS	
Education	Bernzen et al. (2019)	Actual migration; Bangladesh	+(0.01)			
	Codjoe et al. (2017)	Migration intention; Ghana	NS			
	Goldbach (2017)	Actual migration; Indonesia	NS			
	Goldbach (2017)	Actual migration; Ghana	+(0.05–0.01)			
	Gray and Mueller (2012)	Actual migration; Bangladesh	Total −(0.01) Men −(NS–0.05) Women −(0.01)	−(0.01)	NS	
	Haney (2019)	Migration intention; Canada	−(NS–0.10)			
Education of household head	Bohra‐Mishra et al. (2014)	Actual migration; Indonesia		NS		
	Gray and Mueller (2012)	Actual migration; Bangladesh	Total +(NS–0.01) Men NS Women NS	+(NS–0.10)	+(NS–0.05)	
	Mueller et al. (2014)	Actual migration; Pakistan	Men −(0.05) Women NS	Men −(0.05) Women NS	Men NS Women NS	
Gender (female)	Call et al. (2017)	Actual migration; Bangladesh	−(0.01)			
	Codjoe et al. (2017)	Migration intention; Ghana	NS			
	Goldbach (2017)	Actual migration; Indonesia	NS			
	Goldbach (2017)	Actual migration; Ghana	−(NS–0.01)			
	Gray and Mueller (2012)	Actual migration; Bangladesh	+(0.01)	+(0.01)	NS	
	Haney (2019)	Migration intention; Canada	NS			
	Song and Peng (2017)	Migration intention; USA (Florida)	−(0.10)			
Gender of household head (female)	Bohra‐Mishra et al. (2014)	Actual migration; Indonesia		NS		
	Goldbach (2017)	Actual migration; Indonesia	NS			
	Goldbach (2017)	Actual migration; Ghana	+ (NS–0.10)			
	Gray and Mueller (2012)	Actual migration; Bangladesh	Total NS Men NS Women NS	NS	NS	
	Mueller et al. (2014)	Actual migration; Pakistan	Men −(0.01) Women NS	Men −(0.10) Women NS	Men −(0.01) Women NS	
Race (black)	Paxson and Rouse (2008)	Actual migration; USA (Louisiana)	+(NS–0.05)			
	Song and Peng (2017)	Migration intention; USA (Florida)	+(0.05)			
Income/wealth	Bernzen et al. (2019)	Actual migration; Bangladesh	NS			
	Bohra‐Mishra et al. (2014)	Actual migration; Indonesia		NS		
	Call et al. (2017)	Actual migration; Bangladesh	+(0.01)			
	Codjoe et al. (2017)	Migration intention; Ghana	+(0.01)			
	Goldbach (2017)	Actual migration; Indonesia	NS			
	Goldbach (2017)	Actual migration; Ghana	−(0.10–0.05)			
	Gray and Mueller (2012)	Actual migration; Bangladesh	Total +(0.05) Men +(0.01) Women NS	+(NS–0.10)	+(0.10)	
	Haney (2019)	Migration intention; Canada	NS			
	Mueller et al. (2014)	Actual migration; Pakistan	Men NS Women NS	Men NS Women NS	Men NS Women NS	
Unemployed	Goldbach (2017)	Actual migration; Indonesia	−(0.01)			
	Goldbach (2017)	Actual migration; Ghana	+(0.01)			
Farmer as occupation	Bernzen et al. (2019)	Actual migration; Bangladesh	NS			
	Bohra‐Mishra et al. (2014)	Actual migration; Indonesia		NS		
	Codjoe et al. (2017)	Migration intention; Ghana	−(0.01)			
Married	Goldbach (2017)	Actual migration; Indonesia	+(0.05)			
	Goldbach (2017)	Actual migration; Ghana	NS			
	Haney (2019)	Migration intention; Canada	NS			
	Paxson and Rouse (2008)	Actual migration; USA (Louisiana)	NS			
Has children	Gray and Mueller (2012)	Actual migration; Bangladesh	Total −(0.01) Men −(0.01) Women −(0.01)	−(0.01)	−(0.01)	
	Haney (2019)	Migration intention; Canada	NS			
Size or number of children of household	Bohra‐Mishra et al. (2014)	Actual migration; Indonesia		−(0.01)		
	Call et al. (2017)	Actual migration; Bangladesh	−(0.01)			
	Codjoe et al. (2017)	Migration intention; Ghana	NS			
	Goldbach (2017)	Actual migration; Indonesia	Size NS, *N* of children −(0.05)			
	Goldbach (2017)	Actual migration; Ghana	Size +(0.01), *N* of children −(NS–0.10)			
	Gray and Mueller (2012)	Actual migration; Bangladesh	Total NS Men NS Women NS	NS	−(0.10)	
	Mueller et al. (2014)	Actual migration; Pakistan	Men +(0.01) Women +(0.01)	Men +(0.10) Women +(0.01)	Men +(0.05) Women +(0.01)	
	Paxson and Rouse (2008)	Actual migration; USA (Louisiana)	+(NS–0.05)			
Land ownership/access	Bernzen et al. (2019)	Actual migration; Bangladesh	+(NS to 0.01)			
	Gray and Mueller (2012)	Actual migration; Bangladesh	Total NS Men −(0.05) Women NS	NS	−(0.01)	
	Mueller et al. (2014)	Actual migration; Pakistan	Men NS Women −(0.05)	Men NS Women NS	Men NS Women NS	
	Schwaller et al. (2020)	Migration intention; USA (North Carolina)	NS			
Homeownership	Bohra‐Mishra et al. (2014)	Actual migration; Indonesia		−(0.01)		
	Goldbach (2017)	Actual migration; Indonesia	NS			
	Goldbach (2017)	Actual migration; Ghana	NS			
	Haney (2019)	Migration intention; Canada	−(0.05)			
	Paxson and Rouse (2008)	Actual migration; USA (Louisiana)	−(NS–0.05)			
	Schwaller et al. (2020)	Migration intention; USA (North Carolina)	NS			
Time of residence in community	Bernzen et al. (2019)	Actual migration; Bangladesh	NS			
	Boon (2014)	Migration intention; Australia	NS			
	Codjoe et al. (2017)	Migration intention; Ghana	NS			
	Haney (2019)	Migration intention; Canada	NS			
Place attachment	Boon (2014)	Migration intention; Australia	−(0.01)			
	Haney (2019)	Migration intention; Canada	−(NS–0.01)			
Social networks inside place of residence	Bernzen et al. (2019)	Actual migration; Bangladesh	+(0.10)			
	Codjoe et al. (2017)	Migration intention; Ghana	NS			
	Haney (2019)	Migration intention; Canada	NS			
	Paxson and Rouse (2008)	Actual migration; USA (Louisiana)	−(NS–0.05)			
Social networks outside place of residence	Goldbach (2017)	Actual migration; Indonesia	+(0.01)			
	Goldbach (2017)	Actual migration; Ghana	+(NS–0.10)			
Community participation	Bernzen et al. (2019)	Actual migration; Bangladesh	NS			
	Haney (2019)	Migration intention; Canada	+(NS–0.05)			
Migration experience	Goldbach (2017)	Actual migration; Indonesia	NS			
	Goldbach (2017)	Actual migration; Ghana	+(0.10–0.01)			
Risk aversion	Goldbach (2017)	Actual migration; Indonesia	−(0.05)			
	Goldbach (2017)	Actual migration; Ghana	−(0.01)			
Impatience	Goldbach (2017)	Actual migration; Indonesia	−(0.05–0.01)			
	Goldbach (2017)	Actual migration; Ghana	– (0.10–0.05)			
*Macro factors*						
Economic situation (employment, income)	Goldbach (2017)	Actual migration; Indonesia	−(0.05)			
	Gray and Mueller (2012)	Actual migration; Bangladesh	Total+ (0.01) Men NS Women NS	+(0.01)	NS	
Critical infrastructure available (schools, roads, sanitation)	Goldbach (2017)	Actual migration; Indonesia	NS			
	Gray and Mueller (2012)	Actual migration; Bangladesh	Total −(NS–0.05) Men −(NS–0.05) Women NS	−(NS–0.01)	−(NS–0.05)	

*Note*: −/+ is the sign of the effect, significance level in parentheses, NS means effect is not significant. Results displayed in merged cells indicate that the measure encompasses multiple migration destinations.

Abbreviations: CM, migration destination combined or unspecified; INTM, international migration; LIM, long‐distance internal migration; SIM, short‐distance internal migration.

^a^ Goldbach (2017) solely separates results by migration destination for the SLR‐related variables, not for the control variables.

^b^ Only individuals younger than 40 years old are interviewed.
