# Supplementary material for: Anticipating sea‐level rise and human migration: A review of empirical evidence and avenues for future research
Source: Wiley Interdiscip Rev Clim Change. 2021 Nov 12;13(1):e747. doi: 10.1002/wcc.747 (PMC9286789; doi:10.1002/wcc.747)
Supplement: Supplementary file 1 — Appendix S1 Supporting Information [file WCC-13-0-s001.docx]

**Supplementary information**

**S1: Systematic literature search**

*Article inclusion criteria*

The first inclusion criterion states that research should analyze migration intentions or actual migration as the dependent variable. Including studies that investigate migration intentions is relevant for analyzing the potential influence of SLR on migration, because the main effects of SLR lie in the future and, therefore, might not yet be observable through actual or historical migration flows. The second inclusion criterion ensures that research is about the relationship between SLR-related hazards and migration. SLR-related hazards in this context refer to impacts that are expected to become more frequent and intense as a result of SLR (i.e. flooding, erosion, salinization), even though in the study setting they may not yet be attributed to SLR. Studying this relationship can be done in multiple ways, such as by analyzing hazard experience, risk perceptions, or geographically-measured risk. The studies should isolate the effects of SLR-related hazards; studies using composite measures of climate/environmental risk are excluded (e.g. Koubi et al. (2016)). Studies focusing on riverine flooding are included, as SLR through backwater effects can increase the incidence and extent of upstream river flooding (Ikeuchi et al., 2015; Le et al., 2007; Nicholls, 2011), and since such case studies provide important insights into how people respond to flood risk. The third inclusion criterion makes sure that data is collected at the individual or household level. This is important because migration decisions, except for government-forced migration, are generally made by individuals and households, and their characteristics and preferences are pivotal in explaining migration behavior (Black et al., 2011). Regarding the fourth inclusion criterion, the focus is on papers employing multivariate regression analyses. This allows for including multiple independent variables to better approximate causal relationships and estimating the independent effects of SLR-related variables, isolated from the effects of other variables important in the migration decision (Kaczan & Orgill-Meyer, 2020). This focus also facilitates comparison between studies. For all studies, it was essential that they clearly defined the variables included in the analyses, the corresponding data collection process, and the methodologies used.

*Search and screening procedure*

Over the period November and December 2020, we conducted a systematic literature search to search and screen articles for eligibility according to the inclusion criteria described in Section 3. To identify articles, a Web of Science search with the following search term has been performed: *“TS=(sea level* AND ris* OR salin* OR flood* OR inundat* OR erosion) AND TS=(migrat* OR relocat* OR displac* OR retreat* OR mobilit* OR resettl*) AND TS=(survey* OR questionnaire* OR interview* OR experiment* OR empiric* OR perception* OR preference* OR resident* OR household* OR individual*) NOT TS=(oil OR fluid* OR metal* OR electr* OR particle* OR vertical* OR larv*)”.* Only research published since 2000 and in the English language are included in the search. The first part of the search term reflects that the article should be about SLR or SLR-related hazards, the second part that the article should be dealing with migration, and the third part that the article should be of an empirical nature. Using these criteria yielded 9,234 articles. After scanning the results it became clear that many studies dealt with research on oil extraction, fluids, metals, electrochemistry, particles, vertical migration of fish, or larvae. As a consequence, the last part of the search term reflects the exclusion of these articles. This yielded a total of 5,353 articles.


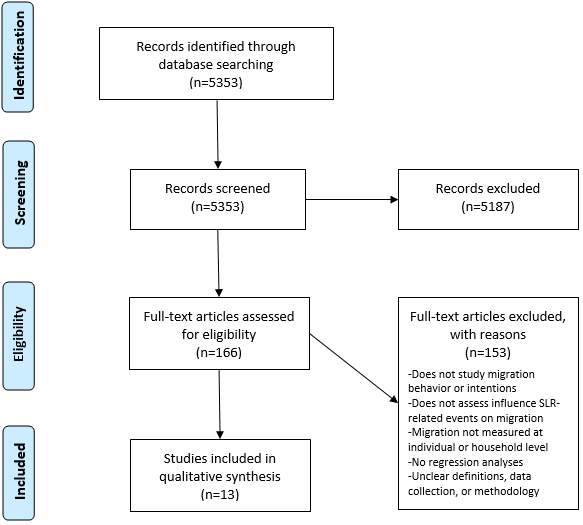


**Figure S1.1:** Article screening process flowchart, adapted from Moher et al. (2009)

These articles were then screened for eligibility based on the four inclusion criteria (see Figure S.1). First, the titles of the 5,353 articles were checked, and articles clearly not dealing with human migration in the context of SLR-related impacts were excluded from consideration. These included, for instance, articles that focused on engineering, medicine, physical hydrology or biology. In the following step, the abstracts of the remaining 443 articles were checked and those not adhering to the inclusion criteria were again excluded. Articles were omitted with caution, and only those clearly not eligible were excluded from consideration. Of the remaining 166 articles, the full text was read. This resulted in the further exclusion of 153 articles and the inclusion of 13 articles in this literature review. Articles excluded at this stage either did not study actual migration or migration intentions, did not assess the influence of SLR-related hazards on migration, did not measure migration at the individual or household level, did not conduct regression analyses, or used unclear data or methodologies. For many articles, more than one of these exclusion criteria applied.

After the Web of Science search and screening process, the references of the 13 included studies were scrutinized with the aim of identifying more eligible studies. In addition, the references of a selection of review articles on the environment-migration nexus were scanned (i.e. Berlemann & Steinhardt, 2017; Black et al., 2011, 2013; Hauer et al., 2020; Kaczan & Orgill-Meyer, 2020; Klaiber, 2014; Penning-Rowsell et al., 2013; Wrathall et al., 2019). Following the same inclusion criteria, this resulted in two more articles to be included in the review.

**References**

Berlemann, M., & Steinhardt, M. F. (2017). Climate Change, Natural Disasters, and Migration—A Survey of the Empirical Evidence. *CESifo Economic Studies*, *63*(4), 353–385. https://doi.org/10.1093/cesifo/ifx019

Black, R., Adger, W. N., Arnell, N. W., Dercon, S., Geddes, A., & Thomas, D. (2011). The effect of environmental change on human migration. *Global Environmental Change*, *21*, S3–S11. https://doi.org/10.1016/j.gloenvcha.2011.10.001

Black, R., Arnell, N. W., Adger, W. N., Thomas, D., & Geddes, A. (2013). Migration, immobility and displacement outcomes following extreme events. *Environmental Science & Policy*, *27*, S32–S43. https://doi.org/10.1016/j.envsci.2012.09.001

Hauer, M. E., Fussell, E., Mueller, V., Burkett, M., Call, M., Abel, K., McLeman, R., & Wrathall, D. (2020). Sea-level rise and human migration. *Nature Reviews Earth & Environment*, *1*(1), 28–39. https://doi.org/10.1038/s43017-019-0002-9

Ikeuchi, H., Hirabayashi, Y., Yamazaki, D., Kiguchi, M., Koirala, S., Nagano, T., Kotera, A., & Kanae, S. (2015). Modeling complex flow dynamics of fluvial floods exacerbated by sea level rise in the Ganges–Brahmaputra–Meghna Delta. *Environmental Research Letters*, *10*(12), 124011. https://doi.org/10.1088/1748-9326/10/12/124011

Kaczan, D. J., & Orgill-Meyer, J. (2020). The impact of climate change on migration: A synthesis of recent empirical insights. *Climatic Change*, *158*(3–4), 281–300. https://doi.org/10.1007/s10584-019-02560-0

Klaiber, H. A. (2014). Migration and household adaptation to climate: A review of empirical research. *Energy Economics*, *46*, 539–547. https://doi.org/10.1016/j.eneco.2014.04.001

Koubi, V., Spilker, G., Schaffer, L., & Bernauer, T. (2016). Environmental Stressors and Migration: Evidence from Vietnam. *World Development*, *79*, 197–210. https://doi.org/10.1016/j.worlddev.2015.11.016

Le, T. V. H., Nguyen, H. N., Wolanski, E., Tran, T. C., & Haruyama, S. (2007). The combined impact on the flooding in Vietnam’s Mekong River delta of local man-made structures, sea level rise, and dams upstream in the river catchment. *Estuarine, Coastal and Shelf Science*, *71*(1–2), 110–116. https://doi.org/10.1016/j.ecss.2006.08.021

Moher, D., Liberati, A., Tetzlaff, J., Altman, D. G., & The PRISMA Group. (2009). Preferred Reporting Items for Systematic Reviews and Meta-Analyses: The PRISMA Statement. *PLoS Medicine*, *6*(7), e1000097. https://doi.org/10.1371/journal.pmed.1000097

Nicholls, R. (2011). Planning for the Impacts of Sea Level Rise. *Oceanography*, *24*(2), 144–157. https://doi.org/10.5670/oceanog.2011.34

Penning-Rowsell, E. C., Sultana, P., & Thompson, P. M. (2013). The ‘last resort’? Population movement in response to climate-related hazards in Bangladesh. *Environmental Science & Policy*, *27*, S44–S59. https://doi.org/10.1016/j.envsci.2012.03.009

Wrathall, D. J., Mueller, V., Clark, P. U., Bell, A., Oppenheimer, M., Hauer, M., Kulp, S., Gilmore, E., Adams, H., Kopp, R., Abel, K., Call, M., Chen, J., deSherbinin, A., Fussell, E., Hay, C., Jones, B., Magliocca, N., Marino, E., … Warner, K. (2019). Meeting the looming policy challenge of sea-level change and human migration. *Nature Climate Change*, *9*(12), 898–901. https://doi.org/10.1038/s41558-019-0640-4
